# Prevalence and determinants of self-reported halitosis in Saudi Arabia: a nationwide cross-sectional study

**DOI:** 10.3389/froh.2026.1900485

**Published:** 2026-07-31

**Authors:** Faisal Mehsen Alali, Bassel Tarakji, Anas Alsalhani, Samer Rastam, Nasser Raqe Alqhtani, Abdullah Saad Alqahtani, Abdullah Bin Nabhan, Faisal S. Alhedyan, Abdullah Almansour, Banna Alnufaiy, Rawda Omar Alghabban, Rayan Awadh Alaonzy, Ahmed Sultan Al-Ateeq, Abdulaziz Hasan Obied, Obaid Khalid Alanazi, Omar Abdullah Almawwi, Mohammad Zakaria Nassani

**Affiliations:** 1 Department of Oral and Maxillofacial Surgery and Diagnostic Sciences, College of Dentistry, Prince Sattam Bin Abdulaziz University, Al Kharj, Saudi Arabia; 2 Department of Histology and Pathology, Faculty of Dentistry, University of Aleppo, Aleppo, Syria; 3 Department of Dentistry, Vision Colleges, Riyadh, Saudi Arabia; 4 Department of Histology and Pathology, Faculty of Dentistry, University of Hama, Hama, Syria; 5 Department of Medicine, Vision Colleges in Riyadh, Riyadh, Saudi Arabia; 6 Department of Preventive Dental Sciences, College of Dentistry, Prince Sattam Bin Abdulaziz University, Al-Kharj, Saudi Arabia; 7 Department of Paediatric Dental Sciences, College of Dentistry, Prince Sattam Bin Abdulaziz University, Alkharj, Saudi Arabia; 8 College of Dentistry, Prince Sattam Bin Abdulaziz University, Al Kharj, Saudi Arabia; 9 Department of Restorative and Prosthetic Dental Sciences, College of Dentistry, Dar Al Uloom University, Riyadh, Saudi Arabia; 10 Department of Removable Prosthodontics, Faculty of Dentistry, University of Aleppo, Aleppo, Syria

**Keywords:** halitosis, oral health, oral hygiene, Saudi Arabia, self-report

## Abstract

**Objectives:**

Halitosis, or oral malodor, is a common condition with important psychosocial consequences. Self-reported halitosis (SRH) reflects perceived oral malodor rather than objectively confirmed halitosis and remains insufficiently studied in Saudi Arabia. This study aimed to determine the prevalence of SRH among Saudi adults and to examine its associations with demographic, systemic, and oral health-related factors.

**Methods:**

A nationwide cross-sectional survey was conducted between February and June 2025 using a 38-item self-administered web-based questionnaire developed from previous literature, reviewed by experts, and pilot-tested before data collection. The questionnaire was distributed via social media platforms and collected data on demographics, systemic health, oral health practices and conditions, and medication use. A total of 1,064 complete responses were analyzed. Descriptive statistics summarized participant characteristics. Chi-square tests examined bivariate associations, and multivariable binary logistic regression identified independent factors associated with SRH.

**Results:**

Among 1,064 participants, 46.9% reported SRH, with the highest prevalence observed among adults aged 31–50 years (55.4%, *p* < 0.0015). Self-reported halitosis was significantly associated with age, self-reported general health, gastritis, long-term sedative intake, and several oral and nasal health indicators (*p* < 0.0015). The multivariate logistic regression model identified self-reported oral health as a significant predictor of SRH. Compared with participants who reported good oral health, those reporting poor oral health were nearly fourteen times as likely to report SRH (OR = 13.97; 95% CI: 7.45–26.22; *p* < 0.001). Brushing teeth also emerged as a significant predictor; participants who brushed their teeth were less likely to report SRH (OR = 0.54; 95% CI: 0.33–0.88; *p* = 0.01). No additional demographic, general health, or oral health variables were significant predictors of SRH in the adjusted model.

**Conclusions:**

SRH is common among Saudi adults and appears to be more closely related to perceived oral health and oral hygiene behaviours than to sociodemographic factors and systemic or nasal conditions assessed in this study. These findings support integrating SRH assessment into oral health promotion and routine dental care, with emphasis on preventive oral hygiene, professional consultation, and culturally appropriate public health strategies. Future studies combining self-reported measures with objective clinical assessment are needed to distinguish perceived from clinically confirmed halitosis and to guide targeted interventions.

## Introduction

Halitosis is commonly described as an unpleasant or offensive odor that may originate from oral or non-oral sources (1). Clinically, halitosis-related complaints should be distinguished according to whether malodor is objectively detectable or only subjectively perceived; the latter may include conditions such as pseudohalitosis or halitophobia, in which perceived malodor is not objectively confirmed (2). In epidemiological research, however, self-reported halitosis (SRH) should be interpreted as a subjective report of perceived bad breath rather than as an objective clinical diagnosis or as a synonym for pseudohalitosis or halitophobia (1, 2). This distinction is important because individuals’ perceptions of oral malodor, rather than objective odor alone, may influence emotional and behavioral responses in social settings (3). Because perceived oral malodor may be associated with anxiety, embarrassment, reduced self-confidence, and impaired social interactions, SRH represents a relevant population-level outcome for understanding the perceived burden of halitosis and its potential care-seeking implications (3, 4).

The etiology of halitosis is multifactorial, although most cases are attributed to intra-oral factors. A recent systematic review reported that 80%–90% of halitosis is caused by intra-oral factors, particularly tongue coating, periodontal diseases, and poor oral hygiene practices, whereas 10%–20% is induced by extra-oral factors associated with systemic diseases (5). Intra-oral malodor is largely related to microbial degradation of organic substrates and the production of volatile sulfur compounds (VSCs), especially in the presence of tongue coating, periodontal inflammation, dental caries, xerostomia, reduced salivary flow, or inadequate oral hygiene (5, 6). Extra-oral and related contributors may include ear, nose, and throat conditions, gastrointestinal disorders, systemic diseases, and medication-related or other conditions associated with xerostomia or reduced salivary flow (5, 6). Accordingly, evaluating SRH in relation to oral hygiene behaviors, perceived oral health status, systemic conditions, and medication use may provide useful insight into the oral hygiene-related, periodontal, salivary, systemic, and medication-related determinants of perceived oral malodor.

Globally, a systematic review and meta-regression analysis estimated the pooled prevalence of halitosis among adolescents and adults at 31.8%, with substantial heterogeneity across studies (1). In Saudi Arabia, available evidence on self-perceived or self-reported halitosis remains comparatively limited. A previous cross-sectional study among adults residing in Riyadh reported a self-perceived halitosis prevalence of 22.8%, identified associations with oral hygiene practices and smoking-related habits, and found that nearly half of affected participants reported an impact on their social life (7). More recent Saudi studies have examined halitosis in specific populations or settings, including young adults undergoing orthodontic treatment, Saudi females, parental knowledge and perception of clinically assessed halitosis in children, and small clinic-based samples assessing objective halitosis-related clinical parameters among dental patients (8–11). These local studies also indicate that self-perceived halitosis may affect social life and interpersonal comfort among affected participants (7, 9). Although these studies provide valuable local evidence, they were limited to single-city samples, sex-specific samples, orthodontic populations, pediatric knowledge and perception outcomes, or small clinic-based samples rather than a large nationwide adult sample addressing SRH and its associated oral and general health-related factors. Therefore, evidence remains limited regarding the prevalence of SRH among Saudi adults and its associations with self-reported oral health, oral hygiene behaviors, systemic conditions, and medication use.

This study aims to investigate the prevalence of self-reported halitosis among Saudi adults and to evaluate its associations with self-reported oral health status, oral hygiene behaviors, systemic conditions, medication use, and related factors. By addressing these associations in a large nationwide adult sample, the study seeks to fill an important gap in the Saudi literature and to provide insight into SRH as a potential indicator of perceived oral health burden and possible unmet care needs. The findings may inform culturally relevant oral health promotion strategies and support integrated approaches that consider both the oral health-related and psychosocial dimensions of perceived oral malodor.

## Materials and methods

### Study design and ethical considerations

This cross-sectional study utilized a web-based questionnaire to explore the prevalence and associated factors of self-reported halitosis (SRH) among Saudi adults. The study was reported in accordance with the Strengthening the Reporting of Observational Studies in Epidemiology (STROBE) guidelines, and all checklist items were carefully addressed (12). Ethical approval was obtained from the Research Ethics Committee at Prince Sattam bin Abdulaziz University, Al Kharj, Saudi Arabia (SCBR-436/2025). All procedures complied with ethical research standards, prioritizing participant confidentiality and voluntary participation.

### Setting and sample

This cross-sectional study was conducted in the Kingdom of Saudi Arabia and targeted Saudi adults aged 18 years and older from different regions of the country. The calculation assumed a hypothesized outcome prevalence of 50% (recommended for unknown population frequencies), an absolute precision of 5%, and a 99% confidence level. This yielded a minimum required sample size of 664 participants. To account for potential non-response and missing data, this number was increased by 10%, resulting in a target sample size of 730. The final dataset contained 1,064 complete responses, exceeding the minimum required sample size and providing an achieved precision of approximately 4.0% at a 99% confidence level, based on the observed SRH prevalence of 46.9%. Participants were recruited using convenience sampling through popular social media platforms such as WhatsApp, Instagram, Snapchat, Twitter, and Facebook (13).

### Questionnaire development

A 38-item, self-administered questionnaire was developed for this study, drawing from previously validated instruments in the literature (7, 14). These studies provided a framework for questions related to oral health practices and conditions, as well as systemic conditions associated with SRH. The questionnaire was structured into five sections:

1. Demographic Characteristics (e.g., age, gender, education, marital status, nationality)
2. Self-reported Halitosis (personal experience)
3. General Health Status (e.g., chronic conditions, general/oral health perception, weight status)
4. Oral Health Practices and Conditions (e.g., brushing, gum bleeding, dry mouth, dental visits)
5. Medication Use (e.g., long-term use of aspirin, corticosteroids, tranquilizers)

Self-reported halitosis (SRH) was assessed using a single dichotomous question: “Did you suffer from halitosis?”, with response options of “Yes” or “No”. Participants who answered “Yes” were classified as having self-reported halitosis. Predictor variables were assessed as self-reported categorical variables according to the response options provided in the questionnaire. Demographic variables were analysed according to their predefined categories, while general and oral health perception variables were classified according to participants’ selected response categories. Systemic conditions were assessed as separate self-reported yes/no items for each condition, and medication-use variables were assessed as self-reported yes/no items regarding the long-term use of selected medications. These variables were included because oral health conditions, extra-oral systemic conditions, and long-term medication use may contribute to oral malodor through oral, gastrointestinal, respiratory, metabolic, salivary, or medication-related pathways. The questionnaire was initially drafted in English, then translated into Arabic by a bilingual specialist, followed by back-translation to ensure linguistic accuracy (15). A panel of four experts in dentistry, oral medicine and public health reviewed the content for validity. A pilot study was subsequently conducted among 50 Saudi adults to evaluate the clarity, comprehensibility, feasibility, and completion time of the questionnaire. The responses obtained during the pilot study were excluded from the final statistical analysis.

### Data collection procedure

The finalized questionnaire was distributed electronically using Google Forms, with the survey link shared via social media platforms. Data collection was conducted over a five-month period, from the beginning of February to the end of June 2025. Participation was voluntary, and electronic informed consent was obtained from all participants prior to survey initiation. To improve response rates, three reminder messages were sent via social media. All survey items were mandatory to ensure complete responses and maintain data quality.

### Reliability assessment

The internal consistency of the oral health and halitosis-related section of the questionnaire was assessed using Cronbach's alpha coefficient, with values ≥0.70 considered acceptable (16). Reliability analysis was performed using responses from the final sample of 1,064 Saudi adults. The oral health and halitosis domain demonstrated acceptable internal consistency, with a Cronbach's alpha coefficient of 0.72, indicating that the questionnaire items were sufficiently reliable for evaluating self-reported halitosis and associated oral health factors in the study population.

### Data analysis

Data was analyzed using IBM SPSS Statistics, Version 26.0 (IBM Corp., Armonk, NY, USA). Descriptive statistics, including counts and percentages, were used to summarize demographic characteristics, self-reported halitosis (SRH) prevalence, self-reported oral health status, systemic conditions, and medication use. Chi-square tests were used to assess bivariate associations between SRH and categorical variables. For the multivariate analysis, a simultaneous forced-entry method (/METHOD = ENTER) was utilized for the binary logistic regression model to identify factors independently associated with SRH. Predictor variables selected for model inclusion encompassed all established conceptual blocks from our questionnaire framework, including demographics (age, gender, education, marital status), general health status, chronic diseases, regular medication intake profiles, oral health conditions, and nasal diseases to control for confounding comprehensively. Prior to interpreting the model parameters, regression diagnostics were performed to assess potential multicollinearity among predictors using Tolerance and Variance Inflation Factor (VIF) metrics from a linear regression framework using identical variable blocks. No significant multicollinearity was observed; all VIF values were well below conservative thresholds, ranging between 1.07 and 2.17 (minimum tolerance = 0.46). Logistic model calibration and overall fit were rigorously evaluated using the Hosmer-Lemeshow goodness-of-fit test, alongside the Nagelkerke pseudo-R2 index. Categorical and ordinal predictors were entered using indicator (dummy) coding with specified baseline reference categories. Odds ratios (ORs) and 95% confidence intervals (CIs) were calculated. A *p*-value <0.05 was considered statistically significant, with Bonferroni correction applied for multiple comparisons (*p* < 0.0015, 0.05/33).

### Bias mitigation

The survey was distributed across multiple social media platforms as an effort to limit potential selection bias and broaden participant reach, including widely used platforms such as WhatsApp. This strategy was considered appropriate in the Saudi context, where WhatsApp has been reported as the most used social media platform for sharing health-related information (17). However, WhatsApp was the predominant recruitment channel, through which most participants received the survey invitation; therefore, the use of multiple platforms should be interpreted as a strategy to increase reach rather than as eliminating selection bias. Response bias was minimized by ensuring participant anonymity, emphasizing the importance of truthful answers, and phrasing questionnaire instructions neutrally to encourage honest responses without leading participants. Measurement bias was addressed by basing the questionnaire on validated studies (7, 14), expert review by a panel of four specialists, and pilot testing, with feedback from the pilot study used to refine question wording and improve clarity. Sampling bias was addressed by targeting varied demographic groups across Saudi regions, including diverse socioeconomic groups, though the convenience sampling method and the predominance of WhatsApp as a recruitment channel may limit generalizability. Overall, these combined measures aimed to enhance the study's reliability and methodological rigor while acknowledging that potential selection bias could not be fully eliminated.

## Results

### Participant characteristics and prevalence of self-reported halitosis

A total of 1,064 adults from Saudi Arabia participated in the study and completed the survey, which is well above the minimum required sample size of 664, thereby ensuring adequate statistical power. WhatsApp was the main method of communication, through which most participants received the invitation (692; 65%). Almost half of the participants (499; 46.9%) reported halitosis. The majority of participants were aged 18–30 years (652; 61.3%). Females outnumbered males (655; 61.6% vs. 409; 38.4%). Most participants had a college or university education (833; 78.3%). With respect to marital status, 542 (50.9%) were single and 482 (45.3%) were married.

Regarding general health status, 632 (59.4%) reported good health, 397 (37.3%) reported fair health, and 35 (3.3%) reported poor health. A total of 170 (16%) reported chronic diseases. In terms of body weight, 558 (52.4%) reported normal weight, 323 (30.4%) were overweight, and 183 (17.2%) were underweight. Gastritis was the most frequently reported disease (169; 15.9%), followed by thyroid problems (98; 9.2%) and diabetes (66; 6.2%). A large proportion of participants reported a family history of diabetes (750; 70.5%). With respect to the regular intake of medications, 311 (29.2%) reported using any medication, 70 (6.6%) reported long-term aspirin use, 48 (4.5%) reported long-term corticosteroid use, and 89 (8.4%) reported long-term sedative use. The sociodemographic and general health characteristics of the participants are summarized in Table 1.

**Table 1:** Sociodemographic and general health characteristics of participants (*n* = 1064).

Characteristic	*n*	%
Self-reported halitosis	499	46.9
Demographics		
Age		
18–30 years	652	61.3
31–50 years	352	33.1
>50 years	60	5.6
Gender		
Male	409	38.4
Female	655	61.6
Education level		
Secondary school or less	231	21.7
College/University	833	78.3
Marital status		
Single	542	50.9
Married	482	45.3
Divorced/Widowed	40	3.8
Survey communication method		
WhatsApp	692	65.0
Snapchat	310	29.1
Other methods	62	5.8
General health status		
Self-reported general health		
Poor general health	35	3.3
Fair general health	397	37.3
Good general health	632	59.4
Self-reported chronic diseases	170	16.0
Self-reported weight status		
Underweight	183	17.2
Normal weight	558	52.4
Overweight	323	30.4
Self-reported chronic systemic diseases and related health conditions		
Kidney disease	37	3.5
Thyroid problems	98	9.2
Liver disease	35	3.3
Gastritis	169	15.9
Lung disease	49	4.6
Diabetes	66	6.2
Family history of diabetes	750	70.5
Regular intake of medications		
Any medication	311	29.2
Long-term aspirin intake	70	6.6
Long-term corticosteroids intake	48	4.5
Long-term sedative intake	89	8.4

### Self-reported oral health and nasal conditions

The oral health status and nasal diseases reported by participants are presented in Table 2. A total of 402 (37.8%) participants reported good oral health, 544 (51.1%) reported fair oral health, and 118 (11.1%) reported poor oral health. The majority (952; 89.5%) reported brushing their teeth, while 326 (30.6%) reported regular dental visits for calculus cleaning. Gum disease was reported by 294 (27.6%) participants, mobile teeth by 187 (17.6%), difficulty chewing food by 149 (14.0%), dental crowns/bridges by 187 (17.6%), dental implants by 108 (10.2%), and dry mouth by 100 (9.4%). Regarding nasal and related conditions, 100 (9.4%) reported chronic tonsillitis, 52 (4.9%) reported chronic pharyngitis, and 483 (45.4%) reported nasal infections such as congestion or runny nose.

**Table 2:** Self-reported oral health and nasal diseases among participants.

Characteristic	*n*	%
Self-reported oral health		
Poor oral health	118	11.1
Fair oral health	544	51.1
Good oral health	402	37.8
Brushes teeth	952	89.5
Regularly visits a dental clinic for calculus cleaning	326	30.6
Excessive bleeding after tooth extraction or wounds	169	15.9
Bleeding gums	311	29.2
Gum disease	294	27.6
Loose/Mobile teeth	187	17.6
Tendency to grind teeth	211	19.8
Difficulty chewing food	149	14.0
Have dental crowns/bridges	187	17.6
Have dental implants	108	10.2
Dry mouth	100	9.4
Chronic tonsillitis	100	9.4
Chronic pharyngitis	52	4.9
Nose infection (congestion or runny nose)	483	45.4

### Bivariate associations between self-reported halitosis and sociodemographic and general health variables

The association between self-reported halitosis and participants’ demographics and general health status is presented in Table 3. Self-reported halitosis was significantly associated with age, being more prevalent among participants aged 31–50 years (195; 55.4%) compared with those aged 18–30 years (280; 42.9%) and those aged >50 years (24; 40.0%) (*p* < 0.0015). A significant association was also observed with self-reported general health status (*p* < 0.0015), with halitosis reported by 25 participants (71.4%) with poor health, 201 (50.6%) with fair health, and 273 (43.2%) with good health. In addition, self-reported halitosis was significantly more common among participants who reported gastritis (108; 63.9% vs. 391; 43.7%) and among those who reported long-term sedative use (57; 64.0% vs. 442; 45.3%) (*p* < 0.0015).

**Table 3:** Association of self-reported halitosis with demographics and general health status.

Characteristic	Self-reported halitosis % (*n*)	*p*-value
Demographics		
Age (years)		**<0** **.** 0015*
18–30	42.9 (280)	
31–50	55.4 (195)	
>50	40.0 (24)	
Gender		**0**.**240**
Male	44.5 (182)	
Female	48.4 (317)	
Education level		**0**.**90**
Secondary school or less	46.3 (107)	
College/University	47.1 (392)	
Marital status		**0**.**004**
Single	41.9 (227)	
Married	52.1 (251)	
Divorced/Widowed	52.5 (21)	
General health status		
Self-reported general health		**<0**.0015*
Poor	71.4 (25)	
Fair	50.6 (201)	
Good	43.2 (273)	
Self-reported chronic diseases		**0**.**53**
No	46.4 (415)	
Yes	49.4 (84)	
Self-reported weight status		**0**.**004**
Underweight	45.9 (84)	
Normal weight	42.8 (239)	
Overweight	54.5 (176)	
Self-reported chronic diseases and related health conditions		
Kidney disease		**0**.**07**
No	46.3 (476)	
Yes	62.2 (23)	
Thyroid problems		**0**.**08**
No	46.1 (445)	
Yes	55.1 (54)	
Liver disease		**0**.**04**
No	46.3 (476)	
Yes	65.7 (23)	
Gastritis		**<0**.0015*
No	43.7 (391)	
Yes	63.9 (108)	
Lung disease		**0**.**03**
No	46.1 (468)	
Yes	63.3 (31)	
Diabetes		**0**.**37**
No	46.5 (464)	
Yes	53.0 (35)	
Family history of diabetes		**0**.**017**
No	41.1 (129)	
Yes	49.3 (370)	
Regular intake of medications		
Any medication		**0**.**93**
No	46.7 (352)	
Yes	47.3 (147)	
Long-term aspirin intake		**0**.**36**
No	46.5 (462)	
Yes	52.9 (37)	
Long-term corticosteroids intake		**0**.**24**
No	46.5 (472)	
Yes	56.3 (27)	
Long-term sedative intake		**<0**.0015*
No	45.3 (442)	
Yes	64.0 (57)	

Asterisks (*) indicate statistical significance at *p* < 0.0015 (*χ*^2^ statistics with Bonferroni adjustment, 0.05/33).

### Bivariate associations between self-reported halitosis and oral health and nasal conditions

Table 4 presents the association between self-reported halitosis and participants’ oral health status and nasal diseases. Self-reported halitosis was significantly associated with self-reported oral health status (*p* < 0.0015), being most prevalent among participants who reported poor oral health (100; 84.7%), followed by those with fair oral health (303; 55.7%) and those with good oral health (96; 23.9%). Significant associations were also observed with several oral health conditions and practices. Self-reported halitosis was more common among participants who did not brush their teeth (70; 62.5% vs. 429; 45.1%; *p* < 0.0015), those reporting excessive bleeding after tooth extraction or wounds (99; 58.6% vs. 400; 44.7%; *p* < 0.0015), bleeding gums (186; 59.8% vs. 313; 41.6%; *p* < 0.0015), gum disease (170; 57.8% vs. 329; 42.7%; *p* < 0.0015), mobile teeth (119; 63.6% vs. 380; 43.3%; *p* < 0.0015), teeth grinding (133; 63.0% vs. 366; 42.9%; *p* < 0.0015), and difficulty chewing (89; 59.7% vs. 410; 44.8%; *p* < 0.0015). Similarly, participants with dental crowns or bridges (117; 62.6% vs. 382; 43.6%; *p* < 0.0015) and those with dental implants (70; 64.8% vs. 429; 44.9%; *p* < 0.0015) reported significantly higher rates of halitosis. Finally, self-reported halitosis was significantly associated with nasal conditions. Participants reporting nasal infections such as congestion or a runny nose had a higher prevalence of halitosis (257; 53.2% vs. 242; 41.7%; *p* < 0.0015).

**Table 4:** Association of self-reported halitosis with oral health status and nasal diseases.

Characteristic	Self-reported halitosis % (*n*)	*p*-value
Self-reported oral health		**<0** **.** 0015*
Poor	84.7 (100)	
Fair	55.7 (303)	
Good	23.9 (96)	
Brushes teeth		**<0**.0015*
No	62.5 (70)	
Yes	45.1 (429)	
Regularly visits a dental clinic for calculus cleaning		**0**.**21**
No	48.2 (356)	
Yes	43.9 (143)	
Excessive bleeding after tooth extraction or wounds		**<0**.0015*
No	44.7 (400)	
Yes	58.6 (99)	
Bleeding gums		**<0**.0015*
No	41.6 (313)	
Yes	59.8 (186)	
Gum disease		**<0**.0015*
No	42.7 (329)	
Yes	57.8 (170)	
Loose/Mobile teeth		**<0**.0015*
No	43.3 (380)	
Yes	63.6 (119)	
Tendency to grind teeth		**<0**.0015*
No	42.9 (366)	
Yes	63.0 (133)	
Difficulty chewing food		**<0**.0015*
No	44.8 (410)	
Yes	59.7 (89)	
Have dental crowns/bridges		**<0**.0015*
No	43.6 (382)	
Yes	62.6 (117)	
Have dental implants		**<0**.0015*
No	44.9 (429)	
Yes	64.8 (70)	
Dry mouth		**0**.**0016**
No	45.3 (437)	
Yes	62.0 (62)	
Chronic tonsillitis		**0**.**0016**
No	45.3 (437)	
Yes	62.0 (62)	
Chronic pharyngitis		**0**.**0027**
No	45.8 (464)	
Yes	67.3 (35)	
Nose infection (congestion or runny nose)		**<0**.0015*
No	41.7 (242)	
Yes	53.2 (257)	

Asterisks (*) indicate statistical significance at *p* < 0.0015 (*χ*^2^ statistics with Bonferroni adjustment, 0.05/33).

### Multivariable predictors of self-reported halitosis

Predictors of self-reported halitosis are presented in Table 5. The multivariate logistic regression model identified self-reported oral health as a significant predictor of halitosis. Compared with participants who reported good oral health, those reporting fair oral health were more than three times as likely to report halitosis (OR = 3.62; 95% CI: 2.62–4.99; *p* < 0.001), while those reporting poor oral health were nearly fourteen times as likely (OR = 13.97; 95% CI: 7.45–26.22; *p* < 0.001). Brushing teeth also emerged as a significant predictor. Participants who brushed their teeth were less likely to report halitosis compared with those who did not (OR = 0.54; 95% CI: 0.33–0.88; *p* = 0.01). No additional demographic, general health, or oral health variables were significant predictors of self-reported halitosis in the adjusted model.

**Table 5:** Predictors and factors associated with self-reported halitosis.

Characteristic	Odds ratio (95% CI)	*p*-value
Demographics		
Age (years)		
18–30	Ref	
31–50	1.22 (0.77–1.92)	0.40
>50	0.78 (0.38–1.62)	0.51
Gender		
Male	Ref	
Female	1.00 (0.74–1.36)	0.99
Marital status		
Single	Ref	
Married	1.35 (0.87–2.09)	0.19
Divorced/Widowed	1.60 (0.70–3.64)	0.27
General health status		
Self-reported general health		
Poor	Ref	
Fair	0.96 (0.37–2.50)	0.93
Good	1.21 (0.46–3.22)	0.70
Self-reported chronic diseases		
No	Ref	
Yes	0.74 (0.47–1.16)	0.18
Self-reported weight status		
Underweight	Ref	
Normal weight	0.93 (0.63–1.37)	0.73
Overweight	1.16 (0.76–1.77)	0.48
Self-reported chronic diseases and related health conditions		
Kidney disease	0.70 (0.27–1.87)	0.48
Thyroid problems	1.09 (0.63–1.88)	0.76
Liver disease	0.84 (0.33–2.14)	0.71
Gastritis	1.31 (0.87–1.98)	0.20
Lung disease	1.47 (0.67–3.22)	0.34
Diabetes	0.93 (0.46–1.91)	0.85
Family history of diabetes	1.21 (0.88–1.65)	0.23
Regular intake of medications		
Any medication	0.94 (0.66–1.34)	0.75
Long-term aspirin intake	0.65 (0.34–1.23)	0.19
Long-term corticosteroids intake	0.75 (0.36–1.57)	0.45
Long-term sedative intake	1.57 (0.89–2.78)	0.12
Oral health status and nasal diseases		
Self-reported oral health		
Good oral health	Ref	
Fair oral health	3.62 (2.62–4.99)	<0.001*
Poor oral health	13.97 (7.45–26.22)	<0.001*
Brushes teeth	0.54 (0.33–0.88)	0.01*
Regularly visits a dental clinic for calculus cleaning	1.09 (0.79–1.50)	0.61
Excessive bleeding after tooth extraction or wounds	1.22 (0.81–1.84)	0.34
Bleeding gums	1.22 (0.87–1.71)	0.25
Gum disease	1.08 (0.76–1.53)	0.66
Loose/Mobile teeth	1.04 (0.69–1.57)	0.86
Tendency to grind teeth	1.32 (0.90–1.96)	0.16
Difficulty chewing food	0.76 (0.48–1.21)	0.24
Have crowns/bridges	1.37 (0.92–2.04)	0.12
Have dental implants	1.28 (0.77–2.11)	0.34
Dry mouth	0.95 (0.56–1.63)	0.86
Chronic tonsillitis	1.17 (0.66–2.06)	0.59
Chronic pharyngitis	1.37 (0.60–3.15)	0.46
Nose infection (congestion or runny nose)	1.28 (0.96–1.71)	0.09

The odds ratio and 95% confidence interval were calculated by a multivariate binary logistic regression model.

*Denotes a significant difference at *p* < 0.05.

## Discussion

This study represents the first large-scale investigation in Saudi Arabia examining the associations between self-reported halitosis, self-reported oral health status, and self-reported systemic conditions. Unlike earlier research limited to specific subgroups (10), it draws on a diverse national sample, improving the population-level relevance of the findings. The large sample size enhances the precision and reliability of the estimates, while the inclusion of participants from different age groups, genders, marital statuses, and educational levels strengthens external validity.

In our study, nearly half of the participants (46.9%) reported self-perceived halitosis, a prevalence that falls within the global range of 30%–50% (1). This proportion is notably higher than the 22.8% reported in Riyadh, Saudi Arabia (7), which may reflect the limitation of being conducted in a single city, as well as the 23.3% observed in Kuwait (18) and the lower figures reported in European populations (20%–30%) (19). By contrast, our findings are comparable with higher prevalence rates documented in parts of Asia and Africa, including 65.9% among Chinese adults (20), 44.9% in Japanese school children (21), and 44.2% in Ethiopian populations (22). Given the psychosocial burden of halitosis, including embarrassment, reduced self-esteem, and impaired social interactions (4, 23), these findings highlight the importance of standardized diagnostic criteria for more reliable international comparisons.

In our study, self-reported poor oral health was strongly associated with halitosis (Table 5). This association is biologically plausible, as intra-oral factors—including periodontal disease, plaque accumulation, and tongue coating—are established drivers of volatile sulfur compound (VSC) production (24, 25). Periodontal signs such as bleeding gums, tooth mobility, and periodontal pockets, together with tongue coating—the primary intra-oral source of oral malodor—have consistently been implicated in halitosis (26, 27). This finding is also consistent with recent Saudi clinical evidence showing that objectively measured halitosis was associated with higher plaque index and DMFT scores (11).

Toothbrushing emerged as a significant protective factor (Table 5). This finding in our results is consistent with evidence that daily plaque control reduces microbial load, tongue coating, and consequent malodor (28, 29). However, routine dental visits for scaling and calculus removal did not show significant protection in our model, possibly due to confounding by indication, as individuals with pre-existing oral problems are more likely to seek dental care. Additionally, participants with crowns, bridges, or implants had higher rates of SRH, consistent with literature showing that poorly maintained prostheses and restorations can promote plaque retention and peri-implant inflammation, exacerbating halitosis (30, 31).

Age was also significantly associated with SRH. Adults aged 31–50 years were more likely to report halitosis compared to younger and older groups (Table 3). This observation is consistent with prior studies (25, 26) and may be explained by heightened psychosocial awareness and anxiety during mid-life (4), cumulative oral health problems (e.g., gingivitis, restorations), and reduced olfactory sensitivity among older adults, which can attenuate self-awareness of malodor. Psychological factors such as social anxiety and olfactory reference syndrome may amplify SRH perception in socially active age groups (32, 33).

Our study identified significant associations between halitosis and gastritis, nasal infection, and long-term sedative use (Tables 3, 4). These findings are consistent with previous research. Helicobacter pylori-associated gastritis has been strongly linked to halitosis (34, 35) while extra-oral factors, including nasal and sinus conditions, have also been identified as important contributors to oral malodor (36). Together, these observations suggest that both gastrointestinal pathology and nasal infections may contribute to the development of halitosis. Collectively, these results underscore the need to consider systemic health conditions and medication history during halitosis assessment and to carefully evaluate patients’ long-term drug use in clinical management.

Building on these findings, the associations observed between SRH and self-reported oral health, toothbrushing, and gastritis should be interpreted within a broader behavioural and biological context rather than as isolated findings. These factors may reflect interacting oral and systemic pathways, including oral hygiene practices, oral microbial activity, periodontal inflammation, salivary conditions, gastrointestinal health, and VSC production. Dietary habits are relevant to these pathways, as a recent scoping review highlighted the potential contribution of dietary patterns, hydration, macronutrient composition, and micronutrient status to the development and management of halitosis (37). Although dietary habits were not assessed in the present study, nutrition may represent an additional unmeasured factor that could help explain part of the observed associations between oral and systemic conditions and SRH. Therefore, future Saudi studies should include nutrition-related variables when evaluating the determinants of self-reported halitosis.

When interpreting these findings, an important methodological consideration is the distinction between self-reported and objectively diagnosed halitosis. The prevalence reported in this study reflects self-reported halitosis rather than clinically confirmed halitosis. Objective methods, including organoleptic assessment and gas chromatography-based tools such as OralChroma™, remain necessary for confirming oral malodor and measuring volatile sulfur compounds (29, 38). Therefore, the observed prevalence should not be interpreted as the true clinical prevalence of objectively diagnosed halitosis. Nevertheless, SRH remains a relevant population-level measure because it captures individuals’ perception of oral malodor, symptom awareness, psychosocial burden, and potential care-seeking needs. This is particularly important because self-perceived oral malodor may influence emotional, behavioural, and social responses, including anxiety, avoidance-related behaviours, impaired social interactions, and broader well-being and quality-of-life concerns (3, 4, 9).

These considerations have important implications for clinical practice and public health policy. For clinicians, routine enquiry about self-reported halitosis may help identify individuals experiencing perceived oral malodor, psychosocial concern, or potential underlying oral or systemic contributors. Such assessment should be accompanied by evaluation of oral hygiene practices, periodontal indicators, prosthetic or restorative factors, nasal symptoms, gastrointestinal complaints, and relevant medication use (39).

For policymakers, the high prevalence of SRH highlights the urgent need for nationwide awareness initiatives to reduce stigma, promote effective oral hygiene practices, and encourage timely professional consultation. Integrating halitosis-related education into oral health promotion strategies and primary healthcare services may help reduce the perceived burden of halitosis and improve help-seeking behaviour (40).

Future research should integrate self-reported measures with objective clinical assessments, including organoleptic scoring and gas chromatography-based methods, to distinguish perceived from clinically confirmed halitosis and to validate population-based findings (29, 38). Longitudinal studies incorporating detailed age measurement and additional behavioural, nutritional, socioeconomic, and clinical variables are also needed to clarify causal pathways between halitosis and oral and systemic health conditions and to guide targeted prevention strategies in Saudi Arabia.

### Strengths and limitations

This study has several strengths. It addresses an important public health issue using a large nationwide sample of Saudi adults. The study was reported in accordance with STROBE recommendations and used multivariable analysis to identify factors independently associated with SRH. In addition, the structured questionnaire was developed from previous literature and pilot-tested before data collection. Together, these features strengthen the methodological transparency and population-level relevance of the findings. Limitations include reliance on self-reported outcomes rather than objective clinical assessment of halitosis, which may overestimate prevalence compared to organoleptic or instrumental measures. However, as discussed above, this approach remains relevant for capturing perceived oral malodor and its psychosocial and care-seeking dimensions. The cross-sectional design precludes causal inference, and convenience sampling via social media may restrict representativeness despite the large and varied sample. Although several platforms were used for recruitment, WhatsApp was the predominant recruitment channel; therefore, individuals who do not regularly use WhatsApp or social media may have been underrepresented, which should be considered when generalizing the findings to the wider Saudi adult population. Age was collected as broad categories rather than as a continuous variable; therefore, the mean age and the exact age of onset of self-reported halitosis could not be assessed. In addition, several potentially important variables, including smoking status, tongue-cleaning practices, dietary habits, socioeconomic factors, and objective periodontal status, were not assessed and may have acted as sources of residual confounding. Future studies should collect age as a continuous variable and incorporate these additional variables to allow more detailed analyses of age-related patterns and determinants of SRH.

## Conclusion

SRH is common among Saudi adults and appears to be more closely related to perceived oral health and oral hygiene behaviours than to sociodemographic factors and systemic or nasal conditions assessed in this study. These findings suggest that SRH should be addressed within oral health promotion and routine dental care, with emphasis on preventive oral hygiene, professional consultation, and culturally appropriate public health strategies. Future studies combining self-reported measures with objective clinical assessment are needed to distinguish perceived from clinically confirmed halitosis and to guide targeted interventions.

## Data Availability

The raw data supporting the conclusions of this article will be made available by the authors, without undue reservation.
