# Supplementary material for: Prevalence and determinants of self-reported halitosis in Saudi Arabia: a nationwide cross-sectional study
Source: Front Oral Health. 2026 Jul 31;7:1900485. doi: 10.3389/froh.2026.1900485 (PMC13473438; doi:10.3389/froh.2026.1900485)
Supplement: Supplementary file 1 [file Supplementaryfile1.docx]

**Self-reported halitosis and related oral conditions**
Halitosis is an oral condition that may be associated with a number of complex factors. The questions that follow, even though they may not seem to be directly related to the mouth, can help to identify factors associated with self-reported halitosis. Therefore, all the questions are important for this research. All answers are of course strictly confidential.

**Do you agree to participate in this survey?**

a. Yes
b. No

**Personal Information**

1. What is your gender?
   a. Female
   b. Male
2. How old are you?
   a. 18-30
   b. 31-50
   c. More than 50
3. What is your level of education?
   a. Primary/Preparatory School
   b. Secondary School
   c. College/University
4. Marital status?
a. Married
b. Single
c. Divorced
d. Widowed
5. What is the communication method through which you received this survey?
   a. Email
   b. Facebook
   c. Twitter
   d. Instagram
   e. WhatsApp
   f. Telegram
   g. Snapchat

**Halitosis (Bad Breath)**

6. Did you suffer from halitosis?
   (a) Yes
   (b) No

**General health**

7. Are you in good health?
   (a) Yes
   (b) No
8. Do you have any chronic diseases?
(a) Yes
(b) No
9. Is your weight normal?

a. Yes

b No

10. Are you overweight?

a. Yes

b. No

11. Are you underweight?
    a. Yes
    b. No
12. Rate your general health
    a. Poor
    b. Fair
    c. Good
13. Rate your oral health
a. Poor
b. Fair
c. Good

**Oral and Dental Health**

14. Have you ever had excessive bleeding after a tooth extraction or from wounds?
    (a) Yes
    (b) No
15. Do you suffer from bleeding gums?
    (a) Yes
    (b) No
16. Do you have loose or wobbly teeth?
    (a) Yes
    (b) No
17. Do you have a tendency to grind your teeth?
    (a) Yes
    (b) No
18. Do you have difficulty chewing food?
    (a) Yes
    (b) No
19. Do you have crowns or bridges?
    (a) Yes
    (b) No
20. Do you have dental implants?
    (a) Yes
    (b) No
21. Do you brush your teeth?
    (a) Yes
    (b) No
22. Have you been told that you have periodontitis (Gum disease) ?
    (a) Yes
    (b) No
23. Have you been told that you have a dry mouth?
    (a) Yes
    (b) No
24. Do you regularly visit a dental clinic to clean calculus?
    (a) Yes
    (b) No
25. Has a doctor ever told you that you have chronic tonsilitis?
    (a) Yes
    (b) No
26. Has a doctor ever told you that you have chronic pharyngitis?
    (a) Yes
    (b) No
27. Did you suffer from a nose infection (congestion or runny nose)?
    (a) Yes
    (b) No

**Chronic Diseases and Health Conditions**

28. Do you suffer from kidney disease?
    (a) Yes
    (b) No
29. Do you suffer from thyroid problems?
    (a) Yes
    (b) No
30. Do you suffer from liver disease?
    (a) Yes
    (b) No
31. Do you suffer from gastritis?
    (a) Yes
    (b) No
32. Do you suffer from lung diseases?
    (a) Yes
    (b) No
33. Do you suffer from diabetes?
    (a) Yes
    (b) No
34. Do you have family relatives with diabetes?
    (a) Yes
    (b) No

**Medication Use**

35. Are you currently taking any medications on a regular basis?
a. Yes
b. No
36. Have you taken an aspirin for long term?
    (a) Yes
    (b) No
37. Have you taken cortisone for long term?
    (a) Yes
    (b) No
38. Have you taken tranquilisers for long term?
    (a) Yes
    (b) No
